# An Exploration of Dutch Dermatologists’ Experience and Satisfaction With Teledermatology: Sociotechnical and Complex Adaptive System Perspective

**DOI:** 10.2196/56723

**Published:** 2024-07-26

**Authors:** Femke van Sinderen, Craig Kuziemsky, Monique W Jaspers, Linda W Peute

**Affiliations:** 1 Department of Medical Informatics Amsterdam UMC location University of Amsterdam Amsterdam Netherlands; 2 Amsterdam Public Health Digital Health Amsterdam Netherlands; 3 Ksyos Health Management Research Amsterdam Netherlands; 4 Office of Research Services MacEwan University Edmonton, AB Canada

**Keywords:** teledermatology, teledermoscopy, provider satisfaction, dermatologist, dermatology, telemedicine, e-health, COVID-19, telehealth, general practitioner, quality, sociotechnical model

## Abstract

**Background:**

Despite the global upscale of teledermatology during the COVID-19 pandemic, persistent barriers, such as the poor anamnesis and photo quality, hinder its effective use in practice. Understanding Dutch dermatologists’ experiences and satisfaction with using the teledermatology system in the Dutch health care system is needed. A holistic evaluation may provide valuable insight to understand how barriers interrelate which is deemed necessary for the innovation of teledermatology in practice.

**Objective:**

Guided by a complex adaptive system perspective, this study aims to understand Dutch dermatologists’ experience and satisfaction with their training, support communication, interaction, and usage of a teledermatology platform of a Dutch digital hospital during the COVID-19 pandemic, uncovering insights to improve teledermatology services for the future.

**Methods:**

A web-based questionnaire was sent in December 2021 to Dutch dermatologists who (1) had an active teledermatology platform account, and (2) responded to a teledermatology consultation between October 1, 2019, and September 30, 2021. The questionnaire consisted of the validated Store-and-Forward Telemedicine Service User-satisfaction Questionnaire (SAF-TSUQ) questionnaire, and new questions regarding; demographics of teledermatologists, the use of teledermatology during the COVID-19 pandemic, the performance of teledermatology by general practitioners (GP), and the role of dermatologists in the teledermatology process. The open-ended questions were analyzed by a grounded theory approach guided by a sociotechnical model and complemented by a complex adaptive system perspective. A panel discussion with 3 dermatologists was performed to provide additional insight into the responses to the questionnaire.

**Results:**

We obtained responses from 25 out of the 249 (10%) invited dermatologists. Overall, dermatologists had a positive experience with teledermatology. Interestingly, teledermatology use frequency remained unaffected by the COVID-19 pandemic. However, the insufficient quality and incompleteness of the clinical content (photos and anamneses information) of the teledermatology consultation impacted the efficiency of the teledermatology workflow. Dermatologists expressed the need for improvement to avoid time-consuming processes or physical referrals. The panel discussion enriched and confirmed the responses, suggesting solutions like mandatory fields for the GPs for a complete anamnesis.

**Conclusions:**

Dutch Dermatologists view teledermatology as a valuable tool to provide access to dermatology care. However, improvements regarding the quality and completeness of the provided clinical content are necessary for the effectiveness and efficiency of the complex teledermatology system in Dutch health care. This could increase both the dermatologists’ satisfaction and the quality of teledermatology services. Managing trade-offs, such as time investments versus image quality, is crucial for teledermatology implementation and should be assessed from a complexity perspective to understand trade-offs and prevent unintended consequences.

## Introduction

Telemedicine is a method to deliver health care remotely by use of digital technologies and is applied in several medical disciplines such as in the dermatology field [1,2]. Through teledermatology, photos and anamnesis information of different types of skin disorders of patients are digitally submitted by a primary care provider and reviewed by a remote dermatologist [3,4]. In the Dutch health care system, this facilitates the guidance of a general practitioner (GP) in primary care by a remote dermatologist from secondary care on the management of the skin disorder. GPs play a central role in the Dutch health care system in coordinating the patients’ health care needs, including referrals to secondary care. Teledermatology has been used since 1995 [5], but challenges still exist worldwide [6,7], including the inability of dermatologists to conduct a full body skin check and palpation of the lesion. Consequently, dermatologists rely on the information provided by the GP for a diagnosis, emphasizing the need for high-quality photos and anamnesis details. This is illustrated by the Danish studies of Vestergaard et al. In these studies, 9.5%-10% of the images were deemed of poor quality, assessed as “useless” due to issues such as out-of-focus images or missing dermoscopic or overview images [4,8]. Another known barrier is reimbursement issues, such as that in the United States, where patients and dermatologists are not always reimbursed for requesting or providing a teledermatology consultation, respectively [9,10].

An increase of dermatology referrals to secondary care in the Netherlands [11,12] underscores the growing demand for the effective use of teledermatology. This surge is partly driven by the aging Dutch population and increasing skin cancer incidence [13,14]. Within the next decade, incidences of basal cell carcinoma are expected to increase [15], potentially leading to a heightened demand for referrals to secondary dermatological care. Despite these barriers, studies demonstrate the potential of teledermatology for general practice in the Netherlands, particularly in preventing unnecessary referrals end minimizing environmental impact [3,16-18].

The COVID-19 pandemic [19] had a massive impact on dermatology practice, as face-to-face care delivery became limited [20]. This led to a global, sudden upscale of teledermatology use to ensure that dermatologists could continue to provide essential dermatology care while still adhering to public health guidelines [21-25]. This required a steep learning curve from some dermatologists as they had to use teledermatology without adequate training or implementation of the service into their practice. These issues have led to increased support requests from dermatologists to the teledermatology provider [23-25]. Despite progress being made, known barriers to effective teledermatology use and implementation still existed after COVID-19 restrictions were stopped. How dermatologists experienced this need and delivery of training, support communication, interaction, and usage of a Dutch teledermatology platform by service providers and whether the COVID-19 pandemic impacted these perceptions positively or negatively is yet unknown. A holistic evaluation may provide valuable insight to understand how barriers interrelate which is deemed necessary for the innovation of teledermatology in practice and to inform strategies to optimize teledermatology services.

Guided by a complex adaptive system perspective, this study aims to understand Dutch dermatologists’ experience and satisfaction with their training, support communication, interaction, and usage of a teledermatology platform of a Dutch digital hospital during the COVID-19 pandemic, uncovering insights to improve teledermatology services for the future [26,27].

## Methods

### The Questionnaire

The Store-and-Forward Telemedicine Service User-satisfaction Questionnaire (SAF-TSUQ) [28], a Dutch validated questionnaire, consists of 29 closed-ended questions within the following themes: Training (eg, offered training to work with teledermatology), Support Communication (eg, preferred way to communicate with Ksyos), Interaction (eg, teledermatology is easy to use), and Use (eg, teledermatology improves access to dermatology care) with the telemedicine platform (Multimedia Appendix 1). The responses are rated on a 5-point Likert scale (1=strongly disagree to 5=strongly agree) with 2 additional options “I do not know” and “not applicable.” On the basis of this questionnaire, the teledermatology service of Ksyos was evaluated [29]. Ksyos is a digital hospital and the largest teledermatology provider in the Netherlands [29].

We added 21 closed- and 6 open-ended questions to tailor the generic SAF-TSUQ questionnaire to specifically address the nuances of teledermatology use. These additional questions were added for the following themes: demographic information of dermatologists, the use of teledermatology (during the COVID-19 pandemic, eg, experience and frequency of use before or during the COVID-19 pandemic), the use of teledermatology by GPs (eg, photo quality), and to evaluate teledermatology as experienced by the dermatologists (eg, not able to perform a full body skin check; Multimedia Appendix 1). All questions were mandatory, except the optional free text fields at the end of each theme. These new questions were discussed in a focus group with teledermatology experts of Ksyos and reviewed by a dermatologist via email.

### Participants

The supplemented questionnaire was sent by email to dermatologists who (1) had an active teledermatology platform account at the moment of sending the questionnaire, and (2) responded to a teledermatology consultation between October 1, 2019, and September 30, 2021.

The questionnaire was sent out in December 2021. Dermatologists gained access to the questionnaire via a unique link in the email. The link became inactive after the questionnaire was filled in so the questionnaire could only be filled in once. This link only allowed tracking if a questionnaire was filled in, thus the individual responses were not visible for the researchers. We sent reminders once to nonresponders 1 week after sending the questionnaire. Informed consent was requested in the questionnaire for the use of their responses for research purposes.

Participation was voluntary and anonymous. Four gift cards were raffled among respondents who completed the questionnaire in a larger study. The LimeSurvey questionnaire tool was used to store the questionnaire and its responses. The Medical Ethical Committee of the Amsterdam University Medical Center (location AMC) provided a waiver.

### Analysis of the Responses

The responses to the closed-ended questions of the SAF-TSUQ questionnaire were analyzed descriptively per questionnaire theme with RStudio.

The Sittig and Singh sociotechnical model was used for the analysis of the open-ended questions [30]. This sociotechnical model has been previously used to understand sociotechnical barriers and facilitators of digital health use after its implementation [31-34], and consists of eight interrelated dimensions: (1) hardware and software; (2) clinical content; (3) human-computer interface; (4) people; (5) workflow and communication; (6) internal organizational policies, procedures and culture; (7) external rules, regulations, and pressures; and (8) system measurement and monitoring (Table 1).

The open-ended questionnaire responses were analyzed by combining an inductive and a deductive approach. First, during a content analysis with an inductive approach, 2 researchers (LWP, Bibiche Groenhuijzen) systematically and independently reviewed the open-ended questionnaire responses to identify patterns and themes using a grounded theory approach [35]. Secondly, a deductive approach was applied to map the identified themes onto the sociotechnical model. The combined deductive and inductive approach allowed for the potential extension of the Sittig and Singh [30] model.

During the content analysis, the data were ordered into discrete responses during the open coding phase. Responses consisting of multiple sentences were split into smaller units of meaning, if necessary. An inductive coding approach was applied by one researcher (LWP) and one coder (Bibiche Groenhuijzen), meaning that the first codes emerged from the prior content analysis. This is an iterative approach whereby the data are reread and the first codes are refined and, if required, new codes can be created. These refined and new codes were (re)applied to the data until saturation of codes was reached and all responses were coded. This final set of codes is referred to as our set of “subcodes.” The subcode “not applicable” was assigned to responses that could not be interpreted by the researchers, and those responses were, therefore, assigned with the maincode “not able to code” and were not assigned to a dimension. The subcodes represented the rich details of the responses. Patterns emerged from these subcodes from which maincodes were formulated. The maincodes represented higher-level categories of the data compared to the subcodes. During axial coding [36,37], sets of subcodes were grouped by one maincode. The open and inductive coding phase resulted in a codebook of sub- and maincodes (Tables S1 and S2 in Multimedia Appendix 2). The coders (Bibiche Groenhuijzen, FvS) independently assigned sub- and maincodes and were blinded to each other’s codes. Continuous discussion meetings took place between the two coders (BG, FvS) and a researcher (LWP) to discuss the content of the responses and to refine the sub- and maincodes. In addition, the dimensions of the sociotechnical model were discussed. Small adaptations to the definition of the dimensions were made to fit them to the teledermatology evaluation. This was done during the inductive phase of coding [30], since at this moment, we got insights into the meaning and application of the dimensions (Table 1).

Finally with a deductive approach, 1 of the 8 interrelated dimensions of the sociotechnical model [30] was independently assigned by the 2 coders to a response. There was a discussion meeting (Bibiche Groenhuijzen, FvS) until consensus was reached regarding the assigned sub- and maincodes and dimensions. A third researcher (LWP) was involved in the discussion meeting if no consensus was reached.

As part of our comprehensive evaluation, we will discuss teledermatology as a complex adaptive system (CAS) [26,27], characterized by several components that interact with each other as part of achieving broader system outcomes. Tenets of a CAS include emergent behavior (system interactions may result in an outcome that is unpredictable) and nonlinearity (a small change in one part of the system may lead to a large change in another part of the system) [38,39]. The components must be meaningfully integrated to achieve desired outcomes such as accurate diagnostic outcomes, improved patient care, and user satisfaction [9,40]. Teledermatology exhibits characteristics of a CAS where users (eg, patients, GPs, dermatologists) are separated by system concepts (eg, time and space), and different technologies (eg, digital systems and photography equipment). A CAS-guided evaluation allows us to understand how various system subcomponents interact within system operations, supplementing the sociotechnical approach by elucidating the relationship between multiple interacting components in a system.

**Table 1:** Definition of the dimensions adapted to the teledermatology situation [30].

Dimension	Definition
Hardware and software	All technical remarks on hardware and the software used on the (teledermatology) consultation platform, for example, (the ease of use of) the photo equipment, (the ease of) uploading photos, and interoperability issues.
Clinical content	All remarks on the (un)structured, textual or numeric data; information, and knowledge that are stored on the (teledermatology) consultation platform. Also remarks on the (feedback of dermatologist on) quality of the photos provided by GP in the consultation.
Human computer interface	All remarks on the software interaction with the user, for example, on the system layout or front-end features.
People	All remarks on individuals that interact with the system or remarks related to training and learnability.
Workflow and communication	All remarks on how teledermatology is used in the workflow, the impact on workload, and the tasks required to provide appropriate care, and communication with the telemedicine provider.
Organizational policies and procedures	All remarks on structures, policies, financials, and procedures of the telemedicine organization that influence technology management.
External rules, regulations, and pressures	All remarks on external factors outside the telemedicine organization that facilitate or impede efforts to design, implement, use, and evaluate technology, as well as remarks indicating that the use of teledermatology by health care providers has changed due to the COVID-19 pandemic.
System measurement and monitoring	All remarks including system availability, its use by stakeholders, its effectiveness, and associated (un)intended consequences (by the COVID-19 pandemic).
Not able to code	All remarks that were insufficiently specific or not comprehensive to be assigned to a dimension. This dimension also includes remarks about the questionnaire itself.

### Dermatologist Panel Discussion

After the questionnaire study, there was an open, unstructured panel discussion with 3 dermatologists (10, 12, and 13 years’ experience with Ksyos teledermatology platform) where one researcher (FvS) presented the questionnaire outcomes. The interpretation and perspectives of the dermatologists on the questionnaire results, and their experience with teledermatology was discussed. The remarks of the dermatologists were noted and directly mapped onto the adapted dimensions (Table 1).

### Ethical Considerations

The Medical Ethical Commission of the Amsterdam University Medical Center granted a waiver stating that the study did not require additional approval.

## Results

### Demographics Respondents

A total of 25 dermatologists (10%) completed the questionnaire, including informed consent (Table 2). We received 3 hard bouncers, 6 out-of-office emails (for an extended period of time), and 2 dermatologists who indicated that they did not want to receive the questionnaire and reminders. The median time to fill in the questionnaire was 11.50 (average 16.49) minutes.

**Table 2:** Demographics respondents.

		Dermatologists, n (%)
**Gender**		
	Male	13 (52)
	Female	12 (48)
**Age (years)**		
	25-34	2 (8)
	35-44	7 (28)
	45-54	6 (24)
	55-64	9 (36)
	≥65	1 (4)
**Computer skills**		
	Excellent	7 (28)
	Good	13 (52)
	Sufficient	5 (20)
	Bad	0 (0)
**Technical knowledge**		
	I am an innovator who is eager to try out new technology	0 (0)
	I am a pioneer and one of the first to experiment with new technology	6 (24)
	I am a frontrunner; if others are adopting new technology, I want to do the same	16 (64)
	I am a laggard and usually one of the last to try out new technology	3 (12)
**How many times using the Ksyos platform**		
	Daily	12 (48)
	Weekly	3 (12)
	Monthly	8 (32)
	A few times per year	2 (8)
**Duration of use of the Ksyos platform**		
	6-12 months	1 (4)
	1-3 years	1 (4)
	3-5 years	4 (16)
	5-10 years	6 (24)
	More than 10 years	13 (52)

### SAF-TSUQ Question Analysis

**Figure 1:**
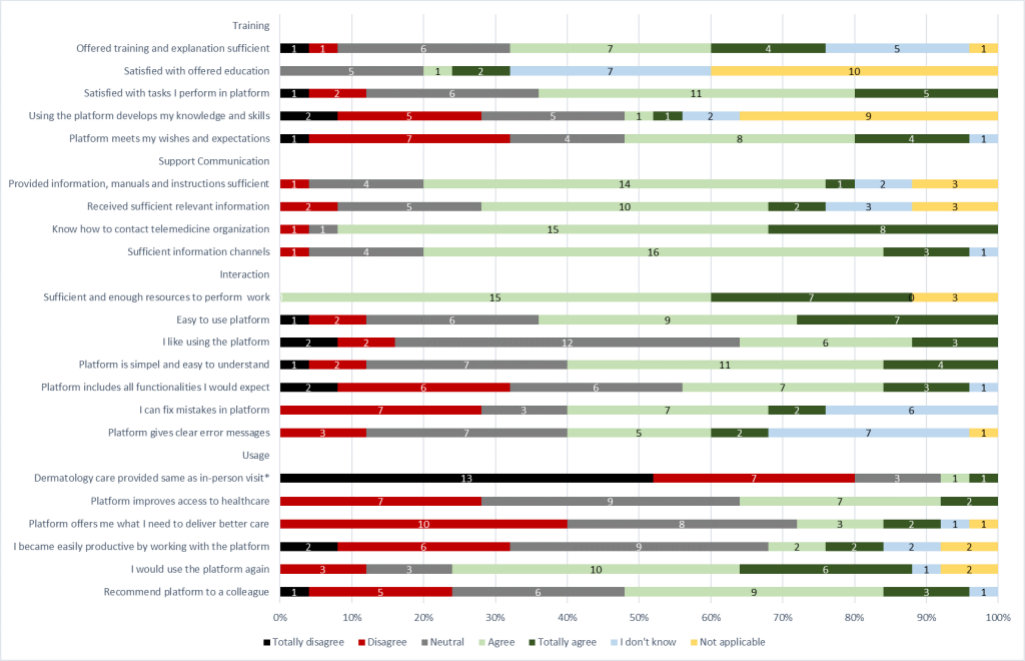
Responses on the Store-and-Forward Telemedicine Service User-satisfaction Questionnaire (SAF-TSUQ) regarding the themes Training, Interaction, Support Communication, and Usage. TD: teledermatology. *Additional explanation in the questionnaire: we meant that a digital consultation could replace a regular consultation.

Overall, dermatologists found that the training, support communication, and information provided by Ksyos was sufficient to work with the platform. However, they were more critical that teledermatology care is not considered the same as providing physical care (Figure 1).

Dermatologists were satisfied with performing the teledermatology tasks. They knew how to contact Ksyos, and if support communication with Ksyos was needed, they preferred to do this via email.

While they found that performing teledermatology did not help them to increase their dermatology knowledge, dermatologists expressed that the platform is easy to use and understandable. Despite this positive feedback, one-third of the dermatologists mentioned that the platform did yet not contain all functionalities to provide teledermatology care (eg, they expressed their preference to provide a diagnosis in a free text field).

The majority of dermatologists acknowledged that teledermatology serves as a valuable medium to provide the GP with advice, recommended the platform and stated that they would use it again.

### Teledermatology During the COVID-19 Pandemic

Some dermatologists reported that teledermatology use slightly increased during the first COVID- 19 wave, compared to prepandemic levels (Table 3). During this time, they noted that sufficient support from Ksyos on how to use teledermatology was received. Dermatologists observed that the type of questions asked by GPs in the teledermatology consultation did not change. They reported difficulties in evaluating teledermatology consultations with poor photo quality and anamnesis information.

**Table 3:** Responses COVID-19 questions.

			Dermatologists, n (%)
**Frequency of using the platform in the first COVID-19 wave vs before the COVID-19 pandemic**			
	More often used	7 (28)	
	Used about as often	17 (68)	
	Used less often	0 (0)	
	Not applicable	1 (4)	
**How often do you use the platform at this moment?**			
	More frequently than before the COVID-19 pandemic	6 (24)	
	About the same as before the COVID-19 pandemic	16 (64)	
	Less frequently than before the COVID-19 pandemic	2 (8)	
	Not applicable	1 (4)	
**Did you receive enough support to perform teledermatology during the COVID-19 pandemic?**			
	Yes	22 (88)	
	No	3 (12)	

### The Sociotechnical Analysis

A codebook consisting of 13 maincodes and 113 subcodes was developed (Tables S1 and S2 in Multimedia Appendix 2). Two coders (FvS, Bibiche Groenhuijzen) had a 65.5% and 71.4% agreement after coding the responses with the sub- and maincodes, respectively. Out of the 247 responses that were coded, the 2 coders were not able to code 35 responses (Table 4). We were able to map all responses on an existing dimension of the Sittig and Sing model (Multimedia Appendix 3).

**Table 4:** Dimensions with related maincodes and exemplary quotes (Q).

Dimension, maincode		Quotes, n	Exemplary Quotes
**System measurement and monitoring**		63	—^a^
	Conditions for use	2	Teledermatology is only suitable for lesions that are on body parts that can be (easily) photographed.
	Effect on care	3	Q1—The patient doesn’t need to visit the hospital and has therefore less risk of a COVID-19 infection.
	General	8	Teledermatology is mostly going fine.
	Learnability	3	I did not learn anything new.
	Need for	3	Teledermatology should be applied on a larger scale.
	Quality of care	22	Q3—The overall picture of the skin is always important when assessing atypical lesions. Q4—It’s about the assessment of only this lesion. I cannot look at other lesions.
	System use	19	Q2—It’s difficult to obtain a diagnosis based on only photos. I miss information about the skin condition and the story of the patient.
	User-friendliness	1	The system is not very user-friendly, although it has improved a bit
	Workload	2	Teledermatology is time-consuming
**Clinical content**		49	—
	Photo quality	35	Q5—The photo quality is insufficient and the specific location of the lesion on the body is sometimes unclear from the photos.
	Quality of care	11	It happens often that a teledermatology consultation contains insufficient information about the patient
	System failure/improvement	2	It would be great if more photos per teledermatology consultation can be added
	Workload	1	It increases my workload if the teledermatology consultation contains photos of insufficient quality
**Organizational policies and procedures**		41	—
	Effect on care	1	There are no restrictions (e.g. certain diagnosis) on when a GP can or cannot send a teledermatology consultation.
	System use	41	Q6—I prefer not to use teledermoscopy, but it is useful for patients for whom it is difficult to visit the hospital
**People**		22	—
	Conditions for use	2	Teledermatology should only be used by specialized GPs
	General	1	The GP is responsible for performing a full-body skin check
	Learnability	2	Q7—The GP can learn from the teledermatology consultations because for me it’s only sharing my knowledge
	Photo quality	1	We should not accept that patients take their own photos due to the low quality
	System use	4	The use of teledermatology during the COVID-19 pandemic did not change
	Training	11	Q8—I provide tips for future consultations. That’s one of my tasks as a specialist; inform the GP what you need to be able to obtain a diagnosis. Q9—GPs should be taught what is needed to make high-quality photographs.
	User-friendliness	1	It is difficult for a lot of patients to upload photos
**Workflow and communication**		21	—
	Conditions for use	2	Certain processes should be faster and more efficient
	Need for	1	Teledermatology is essential
	Quality of care	6	A photo or video cannot replace a physical consultation
	System failure/improvement	1	I use a lot of different platforms which all have different credentials. This should be replaced by one system for digital care.
	System use	7	Sometimes, the patient hasn't even visited the GP, and thus photos are sent by the GP that the patient has taken themselves.
	Workload	3	Teledermatology requires a lot of time compared to a physical consultation
**Human computer interface**		9	—
	System failure/improvement	4	There are too many fixed, mandatory fields
	System use	2	The new layout of the platform has improved
	User-friendliness	3	The platform should be more user-friendly
**Hardware and software**		5	—
	System failure/improvement	5	There should be an app so that I can respond quickly to the GP
**External rules, regulations, and pressures**		2	—
	System use	2	GPs started using teledermatology due to the urgency during the COVID-19 pandemic
Not able to code		35	—
Total		247	—

^a^Not applicable.

### System Measurement and Monitoring

Most dermatologists had a positive experience with teledermatology, especially during the COVID-19 pandemic where they could continue to provide care without seeing the patient at the hospital (Table 4. Q1).

Dermatologists (56%) noted that GPs generally provided complete information in the teledermatology consultations with the required types of photos and anamnesis. However, they were more critical regarding the use of teledermoscopy for diagnosing potential malignant skin lesions. The inability to perform a full body skin check of the patient is a barrier (Table 4. Q2). This underscores the need to receive (overview) pictures of the skin lesion, along with the patient anamnesis information. Dermatologists emphasized the importance of accurately selecting lesions for evaluation, given the ability of GPs to perform a full-body skin check (*quality of care*) (Table 4; Q3, Q4).

### Clinical Content

Dermatologists (44%) expressed their concerns about the low quality of the photos taken by the GP; these are often blurry and assessment (*photo quality*). Also, overview and dermoscopic photos are mostly lacking (*quality of care*), hindering the assessment of the skin type and number of lesions. Consequently, most dermatologists (76%) advise physical referrals (Table 4; Q5).

### Organizational Policies and Procedures

The accurate assessment of potential malignant skin lesions relies heavily on high-quality (dermoscopic) photos [41]. Seven dermatologists considered that teledermoscopy is unsuitable for this purpose, and prefer live dermoscopy for a clearer view of the skin (*system use*). Twelve dermatologists find teledermoscopy suitable and 5 dermatologists preferred not to use teledermoscopy, (Table 4; Q6) indicating different viewpoints for diagnostic suitability for different lesions (*system use*).

### People

Several dermatologists indicate the importance of training GPs in providing high-quality photos and complete patient information (*training,* Table 4; Q9). They believe such training will help GPs understand which information is required for accurate assessment by dermatologists. Dermatologists assert that they feel adequately trained in using teledermatology due to provided instructions, and emphasize that teledermatology does not enhance their dermatological knowledge but assists GPs in diagnosing skin lesions (Table 4; Q7, Q8).

### Workflow and Communication

Three dermatologists find teledermatology more time-consuming than a physical consultation (*workload*), especially with poor-quality photos (*quality of care*). For these reasons, dermatologists prefer a physical consultation as they cannot replace the full body skin check (*quality of care*). Dermatologists believe that teledermatology is, therefore, only useful for dermatologists who have sufficient time. Other dermatologists indicate that teledermatology is indispensable, for example, to monitor patients with a known diagnosis and to supervise the GP.

### Human-Computer Interface

Dermatologists differ in their viewpoint about the usability of the platform. While some believe that improvements have been made in the last years (*system use*), others mention that there are too many fixed, mandatory fields in the teledermatology consultation form which decreases their possibility to enter free text (*System failure/improvement*).

### Hardware and Software

Dermatologists provide suggestions for system improvements, for example, that the photography equipment of the GP should automatically focus to yield better photos, or a mobile app to respond to teledermatology consultations (*System failure/improvement*).

### External Rules, Regulations, and Pressures

Three dermatologists observed that GPs used teledermatology more often during the COVID-19 pandemic than before, driven by the urgency to be able to provide dermatological care.

### Dermatologist Panel Discussion

Comments of the dermatologists mostly matched the questionnaire responses (Table 5). We obtained additional insight into the experiences of dermatologists with teledermatology, since also new findings compared to the questionnaire responses were mentioned. Dermatologists gave possible explanations for critical responses on the use of teledermoscopy provided by dermatologists in the questionnaire (*Organizational policies and procedures, External rules, regulations, and pressures*). There were no comments made regarding the dimension “System measurement and monitoring.”

**Table 5:** Comments panel discussion mapped on the adapted dimensions and compared to questionnaire results.

Dimension	Comments panel discussion	Compared to questionnaire results
Hardware and software	Preference for dedicated fields related to the question and suspected diagnosis of the GP, instead of 1 set of fixed, generic questions in the teledermatology consultation.	Known from questionnaire
Clinical content	The photo quality must be improved.	Known from questionnaire
Human-computer interface	More fields regarding the anamnesis should be mandatory so that the GP will provide more anamnesis information. The provided information by filling in those fields is necessary to be able to diagnose the lesion of the teledermatology consultation.	New result
Human-computer interface	Dermatologists prefer a free text entry field to fill in the diagnosis, instead of a field where a diagnosis is filled in based on a thesaurus. This is too restrictive.	Known from questionnaire
People	GPs must be instructed on the importance of anamnesis information as well as how to obtain high-quality images that dermatologists will require to diagnose the lesion.	Known from questionnaire
Workflow and communication	An option for GPs to request an urgent consultation.	New result
Workflow and communication	Currently, GPs can react to the dermatologists’ response to the teledermatology consultation. GPs mostly use this to thank dermatologists, which is confusing for dermatologists because they expect additional questions.	New result
Organizational policies and procedures	The use of teledermoscopy is discouraged, because the Dutch GP guidelines (“*Nederlands Huisartsen* *Genootschap*”) advice to physically refer patients with suspicious lesions.	New result
External rules, regulations, and pressures	The Dutch Board of Dermatologists (“*Nederlandse Vereniging voor Dermatologie en Venereologie*”) is cautious regarding the application of teledermoscopy for the diagnosis of potential malignant skin lesions, since they believe that there is yet insufficient evidence regarding the diagnostic accuracy of teledermoscopy. As a result, there are different viewpoints on the use of teledermoscopy which discourages the use.	New result
System measurement and monitoring	No comments	N/A^a^

^a^N/A: not applicable.

## Discussion

### Main Findings

Dermatologists showed overall satisfaction with the teledermatology service, and considered it a valuable addition to dermatology care, rather than a replacement of face-to-face consultations. Dermatologists find supervising GPs in patient and lesion management especially valuable. Teledermatology facilitates efficient triage, aiding in prioritizing appointments in secondary care, and thus, streamlines the referral assessment process for dermatologists, ultimately saving time in dermatology practice. Despite reported preferences for face-to-face consultations, dermatologists consistently reported high satisfaction rates with teledermatology [3,42,43]. Teledermatology provides appropriate low-complex diagnostic care at the right place, although acknowledging limitations. The current health care landscape is marked by challenges, including rising health care costs and long hospital waiting times. Teledermatology emerges as a solution to enhance efficient dermatology care [44]. However, barriers were also identified through qualitative analysis of the open-ended responses. Further, this discussion also explores factors that intricately influenced the dermatologists’ experience and satisfaction with the use of teledermatology.

First, dermatologists reported that incomplete anamnesis information or low-quality (dermoscopic) photos (eg, blurry, bad lightning), from GPs hinders an accurate diagnosis, as they heavily rely on this, due to the inability to conduct a full body skin check (*System measurement and monitoring* [45,46]. From the study’s holistic perspective, the trade-off between the convenience of teledermatology (saving patient travel time and less unnecessary physical referrals) and the dermatologists’ preference for physical consultations (depending on lesion complexity) is influenced by the quality of the provided clinical content.

The suboptimal quality of photos and anamnesis information may lead to more time-consuming teledermatology assessments (*Workflow and communication)*, emphasizing the need for adequate clinical content quality. However, this illustrates a trade-off in teledermatology use, namely the balance between the photo quality and the time a GP spent on taking photos. High-quality photos will likely increase the workload of the GP, but will save dermatologists’ time during assessment and improve the dermatologists’ confidence and diagnostic performance [47,48]. In contrast, a consultation with low quality images will likely be considered ineligible for assessment by the dermatologists [3,49]. These barriers were acknowledged during the panel discussion. The poor image quality barrier was already identified in 2011 in the United States [9], making it remarkable that this continues to exist in 2020 in Denmark [4] and in our study. Despite the time-efficiency of teledermatology [3], this study did not assess how quality of the clinical content affects the time needed for assessment by dermatologists.

Teledermatology was already widely implemented in Dutch general practice prior to the pandemic, leading to a more gradual uptake compared to other countries [20]. Dermatologists did not observe a massive increase in the number of teledermatology consultations during or after the pandemic, which indicates its well-established use in the Netherlands. Despite the global forced uptake of teledermatology during the COVID-19 pandemic, both patients and their treating practitioners viewed the experience positively [50].

Our findings demonstrated how barriers impacted different aspects of care delivery. Exclusively analyzing the closed-ended questions of the SAF-TSUQ questionnaire would not have unveiled these findings, as those specifically focused only on the platform usage and omitted other aspects of teledermatology (eg, workload, clinical content). Applying a user-centered approach by surveying the dermatologists gave a better understanding of the dermatologists experiences and satisfaction with using teledermatology. This emphasizes the need for improvements to ensure that teledermatology remains of added value, facilitating its acceptance and implementation in general health care, rather than viewing it solely as a separate digital system [51].

From a sociotechnical CAS perspective, we aimed to understand the impact of interacting system components (eg, photo quality vs workload). While acknowledging that this study is limited in identifying all complex relationships of teledermatology use and that poor photo quality is not a new result, we provide a different perspective on how to think about those barriers and how they present trade-offs in managing these issues. The interrelated nature of digital health implementation means that we cannot view barriers as isolated entities but rather we must think of them as an interrelated system [30]. Our integrated sociotechnical-CAS perspective can be used to study digital health in other settings.

### Future Research

Future research could address the refinement of training methods for GPs to optimize their dermatology knowledge and use of the advanced photography equipment (*People).* The training should be time-efficient and flexible, given the high workload of GPs. We suggest that accreditation would be a nice incentive for participation. Second, automatic focus of camera equipment (*Hardware and software),* and artificial intelligence assisted (image) analysis tools to improve the quality of the provided clinical content should be investigated. The critical attitude of dermatologists toward teledermatology should be investigated. Despite advancements of photography equipment over the past decade and the widespread use of teledermatology, dermatologists remain critical. However, caution is needed when making aforementioned changes in teledermatology systems, since the barriers resulting from this study cannot be evaluated as an isolated entity. Changes could result in unintended consequences on various parts of the system. This underscores the complexity of teledermatology innovation. By looking through a complex system perspective, we could not only identify the barriers but also support the investigation of the consequential impacts on various aspects when modifying one simple factor to a CAS [26,27].

### Strengths and Limitations

We achieved an acceptable response rate of 10%, considering the fact that the questionnaire was distributed during a lockdown. A comparable response rate (13%) was achieved in the study of Kennedy et al [52]. The panel discussion enriched perspectives, thereby balancing diverse viewpoints. This study is part of a larger investigation into teledermatology, wherein also the experiences of GPs were investigated [53]. Through the additional open-ended questions and panel discussion, we were able to provide in-depth insight into the responses of closed-ended questions of the SAF-TSUQ questionnaire. We thus believe that we obtained valid responses. This study was the first step to investigate the experiences of dermatologists with the use of teledermatology in the Netherlands. Follow-up research among a larger group of dermatologists is needed to enrich our results.

Secondly, we used a sociotechnical model as a theoretical framework to thematically analyze the responses on the questionnaire, ensuring a consistency and reliability across the data evaluation process. Through structured coding, we were able to systematically explore the responses, revealing interconnected themes such as that “workload,” which could be related to “Clinical content” (poor photo quality), “Workflow and communication” (time-consuming), and “System measurement and monitoring” (unable to conduct a full body skin check). More information could be gathered from research in practice in addition to the questionnaire responses (eg, observing and interviewing how dermatologists and GPs use the teledermatology service). While various frameworks exist, such as Rogers’ diffusion of innovations theory [54] and Reason’s Swiss Cheese Model [55], they may have a limited scope for the evaluation of complex systems. In this study, we chose the sociotechnical model of Sittig and Singh [30] that integrates technological and sociotechnical (eg, people, processes) factors. This sociotechnical model helped us to examine the experiences of dermatologists with teledermatology services in a systematic manner [56]. Given the fact that this teledermatology service is implemented since 1995 [5], this sociotechnical model accounts for long-term perspectives, allowing for a thorough analysis of the open responses per dimensions of the model.

We experienced the expected limitations of a questionnaire study. Some incomplete responses were excluded. Since this study was the first step in understanding the dermatologists’ experiences with teledermatology, we believe that a questionnaire combined with a panel discussion was an appropriate method. Follow-up research among a larger group of dermatologists within focus groups could provide a deeper understanding of the experiences. Using a sociotechnical model supported a consistent approach, but the sentiment of responses might be lost [57,58]. However, steps were taken to mitigate this through blinded coding and researcher discussions (FvS, LWP, and BG).

Another limitation included the focus on a single teledermatology service which may restrict the generalizability of our findings. It was unknown if the dermatologists also had experiences with other teledermatology services, and avoided this in the questionnaire for clarity purposes. It should be addressed in follow-up research if the experiences of Dutch dermatologists with the Ksyos teledermatology service are shared across different teledermatology services. This could be achieved by investigating a broader range of teledermatology services across different countries through surveying dermatologists or conducting panel discussions, with a similar methodology used in our study. We acknowledge that the teledermatology system in the unique Dutch health care setting, including our results, may not be generalizable to other health care systems. Additionally, only experienced dermatologists responded to the questionnaire, thus a selection bias may exist. Including dermatologists who use teledermatology less frequent in follow-up research could reveal broader perceptions. Finally, the questionnaire did not address reimbursement and legal aspects, which are important considerations for teledermatology adoption [9,10,56].

### Conclusions

Our study sheds light on the use of teledermatology in the Dutch health care system. Dermatologists generally view teledermatology as a valuable tool to provide access to dermatology care as alternative or decision-making prior to a physical consultation. However, their feedback highlights its challenges in providing effective and convenient care via teledermatology. Currently, trade-offs exist between the convenience of saving patient travel time and the preference for a physical consultation, and the need for high-quality photos versus the time spent by GPs on taking pictures. Improving the photo quality and the completeness of the anamnesis information is crucial. Innovative solutions such as artificial intelligence–assisted analysis tools, and not only GP training, are deemed necessary. Complete teledermatology consultations are a prerequisite for dermatologists to provide added value to the GPs and patients. Addressing barriers and implementing solutions will facilitate the continuous use of teledermatology; however, we emphasize the need to address this from a complexity perspective to understand trade-offs as a means of preventing unintended consequences.

## Appendix

Multimedia Appendix 1 The questionnaire sent to dermatologists, including English translation.

Multimedia Appendix 2 Codebook consisting of maincodes and subcodes (Table S1), including the number of dimensions with corresponding maincodes and subcodes (Table S2).

Multimedia Appendix 3 Distribution of coded responses on the dimensions.

## Abbreviations

CAS: complex adaptive system
GP: general practitioner
SAF-TSUQ: Store-and-Forward Telemedicine Service User-satisfaction Questionnaire
