# Supplementary material for: An Exploration of Dutch Dermatologists’ Experience and Satisfaction With Teledermatology: Sociotechnical and Complex Adaptive System Perspective
Source: JMIR Dermatol. 2024 Jul 26;7:e56723. doi: 10.2196/56723 (PMC11316153; doi:10.2196/56723)
Supplement: Multimedia Appendix 1 [file derma_v7i1e56723_app1.docx]

**Appendix** Questionnaire

* Question is mandatory

General questions

Wat is uw leeftijd?*

*Kies één van de volgende mogelijkheden:*

- 18 t/m 24 jaar
- 25 t/m 34 jaar
- 35 t/m 44 jaar
- 45 t/m 54 jaar
- 55 t/m 64 jaar
- 65 jaar of ouder

What is your age?*

*Choose one of the following options:*

- 18-24 years
- 25-34 years
- 35-44 years
- 45-54 years
- 55-64 years
- ≥ 65 years

Wat is uw geslacht?*

*Kies één van de volgende mogelijkheden:*

- Man
- Vrouw
- Overig
- Wil ik niet delen

What is your sex?*

*Choose one of the following options:*

- Male
- Female
- Other
- Prefer not to say

In welke rol/functie bent u betrokken bij [telemedicine organization]?*

*Meerdere antwoorden mogelijk. Selecteer alle mogelijkheden:*

- Cardioloog
- Dermatoloog
- Doktersassistent
- Huisarts
- Longarts
- Oogarts
- Optometrist
- POH(-GGZ/somatiek)
- Psychiater
- (GZ-)psycholoog of psychotherapeut
- Somnoloog
- Specialist ouderengeneeskunde
- Verpleeghuisarts
- Verslavingsarts
- Verpleegkundig specialist
- Zorgmanager
- Anders, namelijk: …..

In which role/function are you involved in at [telemedicine organization]?*

*Multiple answers possible. Select all that apply:*

- Cardiologist
- Dermatologist
- Physician’s assistant
- General Practitioner
- Pulmonologist
- Ophthalmologist
- Optometrist
- Nurse Practitioner (mental health/somatic)
- Psychiatrist
- (GZ-)Psychologist or psychotherapist
- Somnologist
- Specialist geriatric medicine
- Nursing home physician
- Addiction specialist
- Nurse specialist
- Care manager
- Other, …..

Hoe schat u uw computervaardigheden in?*

*Kies één van de volgende mogelijkheden:*

- Slecht
- Voldoende
- Goed
- Uitstekend

How do you rate your computer skills?*

*Choose one of the following options:*

- Poor
- Sufficient
- Good
- Excellent

Hoe zou u uzelf het beste omschrijven?*

*Kies één van de volgende mogelijkheden:*

- Ik ben een innovator en de eerste die nieuwe technologie wil uitproberen
- Ik ben een pionier en één van de eersten die nieuwe technologie uitprobeert
- Ik ben een voorloper, als anderen nieuwe technologie gebruiken, wil ik het ook gaan gebruiken
- Ik ben een achterloper en meestal één van de laatsten die nieuwe technologie uitprobeert
- Ik ben een achterblijver en de laatste die nieuwe technologie uitprobeert

How would you describe yourself?*

*Choose one of the following options:*

- I am an innovator and the first to try out new technology
- I am an early adopter and am one of the first to try out new technology
- I am a part of the early majority. If others use a new technology, I want to start using it as well.
- I am a part of the late majority and am usually one of the last to try out new technology
- I am a laggard and am the last to try out new technology

Maakt u gebruik van het [telemedicine platform] in de praktijk?*

*Meerdere antwoorden mogelijk. Selecteer alle mogelijkheden:*

- Ja, ik verstuur aanvragen en/of consulten en/of onderzoeken naar een specialist
- Ja, ik beoordeel consulten en/of onderzoeken
- Ja, ik ontvang alleen medische uitslagen van [telemedicine organization] in het Huisarts Informatie Systeem (HIS)
- Ja, ik log in om uitslagen te bekijken
- Ja, ik verstuur online behandelmodules en/of vragenlijsten naar de patiënt
- Nee
- Weet ik niet
- Ja, anders

Do you use the [telemedicine platform] in practice?*

*Multiple answers possible. Select all that apply:*

- Yes, I send requests and/or consultations and/or examinations to a specialist
- Yes, I assess consultations and/or examinations
- Yes, I only receive medical results from [telemedicine organization] in the GP information system
- Yes, I log in to view medical results
- Yes, I send web-based treatment modules and/or questionnaires to the patient
- Nee
- I do not know
- Yes, other…

Welke dienst(en) gebruikt u van [telemedicine organization]?*

*Meerdere antwoorden mogelijk. Selecteer alle mogelijkheden:*

- Cardiologie
- Dermatologie
- GGZ
- Laboratorium aanvragen
- Oogheelkunde
- Pulmonologie
- Slaap
- Geen van bovenstaande
- *Weet ik niet*

Which service(s) do you use from [telemedicine organization]?*

*Multiple answers possible. Select all that apply:*

- Cardiology
- Dermatology
- Mental health
- Laboratory requests
- Ophthalmology
- Pulmonology
- Sleep
- None of the above
- *I do not know*

Hoe vaak gebruikt u het [telemedicine platform] in de praktijk?*

*Kies één van de volgende mogelijkheden:*

- Dagelijks
- Wekelijks
- Maandelijks
- Een aantal keer per jaar
- Nooit

How often do you use the [telemedicine platform] in practice?*

*Choose one of the following options:*

- Daily
- Weekly
- Monthly
- A few times in a year
- Never

Hoe lang werkt u al met het [telemedicine platform]?*

*Kies één van de volgende mogelijkheden:*

- Minder dan een half jaar
- 6-12 maanden
- 1-3 jaar
- 3-5 jaar
- 5-10 jaar
- Meer dan 10 jaar

How long have you been working with the [telemedicine platform]?*

*Choose one of the following options:*

- <6 months
- 6-12 months
- 1-3 years
- 3-5 years
- 5-10 years
- >10 years

Overige opmerkingen

Other comments

COVID-19 pandemic

De volgende vragen hebben betrekking op de digitale zorg gedurende de corona pandemie. De eerste corona golf definiëren we als de start van corona (periode maart – mei 2020).

The following questions are related to digital care during the corona pandemic. We define the first corona pandemic as the start of corona (period March – May 2020).

Hoe vaak heeft u het [telemedicine platform] gebruikt in de eerste corona golf vergeleken met de periode voor de corona pandemie?*

*Kies één van de volgende mogelijkheden:*

- Minder vaak gebruikt
- Ongeveer even vaak gebruikt
- Vaker gebruikt
- *Niet van toepassing*

How often have you used the [telemedicine platform] during the first corona wave compared with the period before the corona pandemic?*

*Choose one of the following options:*

- Less often
- Approximately as often
- More often
- *Not applicable*

Hoe vaak gebruikt u het [telemedicine platform] nu?*

*Kies één van de volgende mogelijkheden:*

- Minder vaak dan voor de corona pandemie
- Ongeveer even vaak als voor de corona pandemie
- Vaker dan voor de corona pandemie
- *Niet van toepassing*

How often do you use the [telemedicine platform] currently?*

*Choose one of the following options:*

- Less often than before the corona pandemic
- Approximately as often as before the corona pandemic
- More often than before the corona pandemic
- *Not applicable*

Ontving u voldoende ondersteuning voor het uitvoeren van digitale zorg tijdens de corona pandemie?*

*Kies één van de volgende mogelijkheden:*

- Ja
- Nee

Did you receive sufficient support for carrying out digital care during the corona pandemic?*

*Choose one of the following options:*

- Yes
- No

*Indien “Nee”:*

Wat zou volgens u aan de ondersteuning verbeterd kunnen worden bij een volgende golf en/of pandemie?*

*If “No”:*

What do you think could be improved about support in the event of another wave and/or pandemic?*

Wat heeft u geleerd tijdens de corona pandemie over de toepassing van digitale zorg?*

What have you learned during the corona pandemic about the application of digital care?*

Overige opmerkingen

Other comments

Training

|  | Helemaal oneens | Oneens | Neutraal | Eens | Helemaal eens | *Weet ik niet* | *Niet van toepassing* |
| --- | --- | --- | --- | --- | --- | --- | --- |
| De training en uitleg die door [telemedicine organization] wordt aangeboden zijn voldoende om het [telemedicine platform] te kunnen gebruiken in mijn werkzaamheden* | ⃝ | ⃝ | ⃝ | ⃝ | ⃝ | ⃝ | ⃝ |
| Ik ben tevreden met de mogelijkheden voor bij- en/of nascholing die door [telemedicine organization] worden aangeboden* | ⃝ | ⃝ | ⃝ | ⃝ | ⃝ | ⃝ | ⃝ |
| Ik ben tevreden met de taken die ik uitvoer binnen mijn functie in het [telemedicine platform]* | ⃝ | ⃝ | ⃝ | ⃝ | ⃝ | ⃝ | ⃝ |
| Door het [telemedicine platform] te gebruiken ben ik in staat om mijn kennis en vaardigheden genoeg te ontwikkelen* | ⃝ | ⃝ | ⃝ | ⃝ | ⃝ | ⃝ | ⃝ |
| Ik ben van mening dat het [telemedicine platform] aan mijn wensen en verwachtingen voor digitale zorgverlening voldoet* | ⃝ | ⃝ | ⃝ | ⃝ | ⃝ | ⃝ | ⃝ |

Met taken wordt bedoeld het versturen van aanvragen en/of consulten en/of onderzoeken naar een specialist, het beoordelen van consulten/onderzoeken, het ontvangen of bekijken van medische uitslagen, het versturen van online behandelmodules en/of vragenlijsten naar de patiënt, etc.

Met “kennis en vaardigheden ontwikkelen” wordt bedoeld in hoeverre u digitale en medische kennis verkrijgt door te werken met het telemedicine platform.

|  | Strongly disagree | Disagree | Neutral | Agree | Strongly agree | *I do not know* | *Not applicable* |
| --- | --- | --- | --- | --- | --- | --- | --- |
| The training and explanation offered by [telemedicine organization] are sufficient to be able to use the [telemedicine platform] in my work* | ⃝ | ⃝ | ⃝ | ⃝ | ⃝ | ⃝ | ⃝ |
| I am satisfied with the possibilities for additional and/or continuing education offered by [telemedicine organization]* | ⃝ | ⃝ | ⃝ | ⃝ | ⃝ | ⃝ | ⃝ |
| I am satisfied with the tasks I perform within my profession in the [telemedicine platform]* | ⃝ | ⃝ | ⃝ | ⃝ | ⃝ | ⃝ | ⃝ |
| By using the [telemedicine platform] I am able to develop my knowledge and skills adequately* | ⃝ | ⃝ | ⃝ | ⃝ | ⃝ | ⃝ | ⃝ |
| I believe that [telemedicine platform] meets my wishes and expectations for telemedicine* | ⃝ | ⃝ | ⃝ | ⃝ | ⃝ | ⃝ | ⃝ |

*‘Tasks’ refers to sending requests and/or consultations and/or examinations to a specialist, assessing consultations and/or examinations, receiving or viewing medical results, sending web-based treatment modules and/or questionnaires to the patient, etc.*

*‘Developing knowledge and skills’ refers to the extent to which you acquire digital medical knowledge by working with the [telemedicine platform].*

Overige opmerkingen

Other comments

Support Communication

Op welke manier(en) communiceert u het liefst met [telemedicine platform]?*

*Meerdere antwoorden mogelijk. Selecteer alle mogelijkheden:*

- E-mail
- Telefoon
- Via een accountmanager
- Via een chatfunctie
- Anders, namelijk…

Hoe wilt u op de hoogte gehouden worden over [telemedicine platform]?* *Meerdere antwoorden mogelijk*. *Selecteer alle mogelijkheden:*

- E-mail
- LinkedIn
- Twitter
- Facebook
- Website
- In het [telemedicine platform] direct na het inloggen
- Ik wil niet op de hoogte gehouden worden
- Anders, namelijk…

In which way(s) do you prefer to communicate with [telemedicine platform]?* *Multiple answers possible. Select all that apply:*

- E-mail
- Phone
- Via an accountmanager
- Via a chat function
- Other,…

How would you like to stay informed about [telemedicine platform]?* *Multiple answers possible. Select all that apply:*

- E-mail
- LinkedIn
- Twitter
- Facebook
- Website
- In [telemedicine platform] directly after logging in
- I do not want to be kept informed
- Other,…

|  | Helemaal oneens | Oneens | Neutraal | Eens | Helemaal eens | *Weet ik niet* | *Niet van toepassing* |
| --- | --- | --- | --- | --- | --- | --- | --- |
| De informatie en handleidingen die vanuit [telemedicine organization] aan mij worden verstrekt en de instructies die ik heb ontvangen om mijn werk uit te voeren zijn voldoende* | ⃝ | ⃝ | ⃝ | ⃝ | ⃝ | ⃝ | ⃝ |
| Ik ontvang voldoende relevante informatie met betrekking tot de besluiten, projecten en activiteiten van [telemedicine organization] die invloed op mij kunnen hebben* | ⃝ | ⃝ | ⃝ | ⃝ | ⃝ | ⃝ | ⃝ |
| Ik weet op welke manier ik contact kan opnemen met [telemedicine organization] indien ik vragen heb over mijn werk voor [telemedicine organization]* | ⃝ | ⃝ | ⃝ | ⃝ | ⃝ | ⃝ | ⃝ |
| Er zijn genoeg informatie kanalen voor suggesties of klachten bij [telemedicine organization]* | ⃝ | ⃝ | ⃝ | ⃝ | ⃝ | ⃝ | ⃝ |

|  | Strongly disagree | Disagree | Neutral | Agree | Strongly agree | *I do not know* | *Not applicable* |
| --- | --- | --- | --- | --- | --- | --- | --- |
| The information and manuals that are provided to me by [telemedicine organization] and the instructions given to perform my work are sufficient* | ⃝ | ⃝ | ⃝ | ⃝ | ⃝ | ⃝ | ⃝ |
| I receive sufficient relevant information regarding any decisions, projects and activities of [telemedicine organization] that may affect me* | ⃝ | ⃝ | ⃝ | ⃝ | ⃝ | ⃝ | ⃝ |
| I know how to contact [telemedicine organization] if I have questions about my work for [telemedicine organization]* | ⃝ | ⃝ | ⃝ | ⃝ | ⃝ | ⃝ | ⃝ |
| There are sufficient information channels for suggestions or complaints at [telemedicine organization]* | ⃝ | ⃝ | ⃝ | ⃝ | ⃝ | ⃝ | ⃝ |

Overige opmerkingen

Other comments

Interaction telemedicine platform

|  | Helemaal oneens | Oneens | Neutraal | Eens | Helemaal eens | *Weet ik niet* | *Niet van toepassing* |
| --- | --- | --- | --- | --- | --- | --- | --- |
| Ik heb genoeg en de juiste middelen (zoals computers, holterkastjes, gebruiksvoorwerpen, etc.) om mijn (dagelijkse) werk voor [telemedicine organization] uit te kunnen voeren* | ⃝ | ⃝ | ⃝ | ⃝ | ⃝ | ⃝ | ⃝ |
| Het is gemakkelijk om het [telemedicine platform] te gebruiken* | ⃝ | ⃝ | ⃝ | ⃝ | ⃝ | ⃝ | ⃝ |
| Ik vind het prettig om het [telemedicine platform] te gebruiken* | ⃝ | ⃝ | ⃝ | ⃝ | ⃝ | ⃝ | ⃝ |
| Het [telemedicine platform] is simpel en gemakkelijk te begrijpen* | ⃝ | ⃝ | ⃝ | ⃝ | ⃝ | ⃝ | ⃝ |
| Het [telemedicine platform] bevat alle functionaliteiten die ik zou verwachten* | ⃝ | ⃝ | ⃝ | ⃝ | ⃝ | ⃝ | ⃝ |
| Wanneer ik binnen het [telemedicine platform] een fout maak kan ik deze gemakkelijk en snel herstellen* | ⃝ | ⃝ | ⃝ | ⃝ | ⃝ | ⃝ | ⃝ |
| Het [telemedicine platform] geeft foutmeldingen die duidelijk vermelden hoe ik problemen kan oplossen* | ⃝ | ⃝ | ⃝ | ⃝ | ⃝ | ⃝ | ⃝ |

|  | Strongly disagree | Disagree | Neutral | Agree | Strongly agree | *I do not know* | *Not applicable* |
| --- | --- | --- | --- | --- | --- | --- | --- |
| I have sufficient and appropriate resources (such as computers, holterdevices, equipment, etc.) to perform my (daily) work for [telemedicine organization]* | ⃝ | ⃝ | ⃝ | ⃝ | ⃝ | ⃝ | ⃝ |
| It is easy to use the [telemedicine platform]* | ⃝ | ⃝ | ⃝ | ⃝ | ⃝ | ⃝ | ⃝ |
| I like using the [telemedicine platform]* | ⃝ | ⃝ | ⃝ | ⃝ | ⃝ | ⃝ | ⃝ |
| The [telemedicine platform] is simple and easy to understand* | ⃝ | ⃝ | ⃝ | ⃝ | ⃝ | ⃝ | ⃝ |
| The [telemedicine platform] includes all functionalities I would expect* | ⃝ | ⃝ | ⃝ | ⃝ | ⃝ | ⃝ | ⃝ |
| Whenever I make a mistake within the [telemedicine platform] I can easily and quickly fix it* | ⃝ | ⃝ | ⃝ | ⃝ | ⃝ | ⃝ | ⃝ |
| The [telemedicine platform] provides error messages that clearly state how I can fix problems* | ⃝ | ⃝ | ⃝ | ⃝ | ⃝ | ⃝ | ⃝ |

Overige opmerkingen

Other comments

|  | Helemaal oneens | Oneens | Neutraal | Eens | Helemaal eens | *Weet ik niet* | *Niet van toepassing* |
| --- | --- | --- | --- | --- | --- | --- | --- |
| Het [telemedicine platform] verbetert de toegang tot de gezondheidszorg* | ⃝ | ⃝ | ⃝ | ⃝ | ⃝ | ⃝ | ⃝ |
| Het [telemedicine platform] biedt mij wat ik nodig heb om betere zorg te leveren | ⃝ | ⃝ | ⃝ | ⃝ | ⃝ | ⃝ | ⃝ |
| Ik werd snel productief door het werken met het [telemedicine platform]* | ⃝ | ⃝ | ⃝ | ⃝ | ⃝ | ⃝ | ⃝ |
| Ik ben van mening dat de dermatologie zorg die via het [telemedicine platform] wordt geleverd hetzelfde is als een regulier consult^+^* | ⃝ | ⃝ | ⃝ | ⃝ | ⃝ | ⃝ | ⃝ |
| Ik ben van plan het [telemedicine platform] nogmaals te gebruiken* | ⃝ | ⃝ | ⃝ | ⃝ | ⃝ | ⃝ | ⃝ |
| Ik zou het [telemedicine platform] aanraden aan een collega* | ⃝ | ⃝ | ⃝ | ⃝ | ⃝ | ⃝ | ⃝ |

Use of telemedicine platform

^+^Hiermee bedoelen we vindt u dat een digitaal consult een regulier consult kan vervangen?

|  | Strongly disagree | Disagree | Neutral | Agree | Strongly agree | *I do not know* | *Not applicable* |
| --- | --- | --- | --- | --- | --- | --- | --- |
| The [telemedicine platform] improves access to healthcare services* | ⃝ | ⃝ | ⃝ | ⃝ | ⃝ | ⃝ | ⃝ |
| The [telemedicine platform] offers me what I need to deliver better care* | ⃝ | ⃝ | ⃝ | ⃝ | ⃝ | ⃝ | ⃝ |
| I quickly became productive while working with the [telemedicine platform]* | ⃝ | ⃝ | ⃝ | ⃝ | ⃝ | ⃝ | ⃝ |
| I believe that the dermatology care provided through the [telemedicine platform] is the same as an in-person consultation^+^* | ⃝ | ⃝ | ⃝ | ⃝ | ⃝ | ⃝ | ⃝ |
| I would use the [telemedicine platform] again* | ⃝ | ⃝ | ⃝ | ⃝ | ⃝ | ⃝ | ⃝ |
| I would recommend the [telemedicine platform] to a colleague* | ⃝ | ⃝ | ⃝ | ⃝ | ⃝ | ⃝ | ⃝ |

^+^We mean do you think that a digital consultation can replace a regular consultation?

Overige opmerkingen

Other comments

The experience of the dermatologists during the teledermatology process

In onderstaande vragen wordt met “digitale dermatologie consultatie” het reguliere en dermatoscopie consult bedoeld.

Wat zijn uw ervaringen met het gebruik van het digitale dermatologie consult (regulier, dermatoscopie) tijdens de corona pandemie?* Kies één van de volgende mogelijkheden:

- Heel negatief
- Negatief
- Neutraal
- Positie
- Heel positief

*Licht uw ervaring toe*

Wat heeft u geleerd van het gebruik van het digitale dermatologie consult (regulier, dermatoscopie) tijdens de corona pandemie?*

Zetten de verwijzers volgens u het reguliere digitale dermatologie of dermatoscopie consult correct in voor de verschillende soorten huidafwijkingen?* *Bijvoorbeeld: een huisarts stuurt een gepigmenteerde laesie in middels een algemeen consult/zonder dermatoscopische foto’s, of een huisarts stuurt een eczeem in middels een dermatoscopie consult/met dermatoscopische foto’s*

- *Ja*
- *Nee*

Licht toe waarom de verwijzers het volgens u wel/niet correct inzetten

Wat doet u in het algemeen bij twijfel van uw diagnose na beoordeling van de foto(’s) van een digitaal dermatologie of dermatoscopie consult?* Kies één van de volgende mogelijkheden:

- *Ik twijfel nooit*
- *Ik geef geen diagnose*
- *Ik adviseer de huisarts de patiënt te verwijzen*
- *Anders, namelijk….*

Heeft u een training gehad over het beoordelen van digitale (dermatoscopische) foto’s?* *Meerdere antwoorden mogelijk. Selecteer alle mogelijkheden:*

- Ja, ik heb dit geleerd tijdens mijn opleiding tot dermatoloog
- Ja, ik heb een (aanvullende) cursus gevolgd
- *Welke (aanvullende) cursus heeft u gevolgd?**
- Ja, ik heb dit geleerd door het opdoen van praktijkervaring
- Nee

“Digital dermatology consultation” refers to both regular and dermatoscopy digital consultations.

What are your experiences with the use of the digital dermatology consultation (regular, dermatoscopy) during the corona pandemic?* *Choose one of the following options:*

- Very negative
- Negative
- Neutral
- Positive
- Very positive

*Explain your experience*

What have you learned from the use of digital dermatology consultations (regular, dermatoscopy) during the COVID-19 pandemic?*

Do you believe referring physicians correctly utilize regular digital dermatology or dermatoscopy consultations for different types of skin conditions?* *For example, a general practitioner sends in a pigmented lesion through a regular consultation/without dermatoscopic photos, or a general practitioner sends in eczema through a dermatoscopy consultation/with dermatoscopic photos.*

- Yes
- No

*Please explain why you think referring physicians do or do not use it correctly.*

What do you generally do when you have doubts about your diagnosis after reviewing the photo(s) from a digital dermatology or dermatoscopy consultation?* *Choose one of the following options:*

- I never have doubts
- I do not provide a diagnosis
- I advise the general practitioner to refer the patient
- Other,...

Have you received training on assessing digital (dermatoscopic) photos?* *Multiple answers possible. Select all options that apply:*

- Yes, I learned this during my dermatologist training
- Yes, I have taken an (additional) course
- Which (additional) course did you take?*
- Yes, I learned this through practical experience
- No

Vindt u over het algemeen dat de kwaliteit van ingestuurde (dermatoscopische) foto’s voldoende is?*

*Kies één van de volgende mogelijkheden:*

- Ja
- Nee

Licht toe waarom u dit wel/niet voldoende vindt

Digitale dermatoscopie consultatie is geschikt voor …

*Denk aan patiënt populatie, type laesies** [open vraag]

Digitale dermatoscopie consultatie is niet/minder geschikt voor *Denk aan patiënt populatie, type laesies** [open vraag]

In hoeverre gaat u ervan uit dat er een totale lichaamsinspectie heeft plaats gevonden bij de huisarts voor aanvang van het digitale dermatoscopie consult?*

*Kies één van de volgende mogelijkheden:*

- Ik ga ervan uit dat dit altijd is gebeurd
- Ik ga ervan uit dat dit soms is gebeurd
- Ik ga ervan uit dat dit niet altijd is gebeurd

Vindt u het zelf niet kunnen uitvoeren van een totale lichaamsinspectie een drempel van het digitale dermatoscopie consult?* *Kies één van de volgende mogelijkheden:*

- Ja
- Nee
- Licht toe waarom u dit wel/niet een drempel vindt

Welke dermatoscopische techniek heeft uw voorkeur bij gebruik van digitale dermatoscopie consulten?*

*Kies één van de volgende mogelijkheden:*

- Contact-dermatoscopie
- Gepolariseerde dermatoscopie
- Beide

Waar ziet u verbetermogelijkheden om een digitaal dermatologie consult optimaal te kunnen beoordelen?*

Overige opmerkingen

Do you generally find the quality of the submitted (dermatoscopic) photos to be sufficient?* *Choose one of the following options:*

- Yes
- No

Please explain why you find this sufficient or insufficient

Digital dermatoscopy consultation is suitable for...

*Think about patient population, types of lesions**

Digital dermatoscopy consultation is not/less suitable for...

*Think about patient population, types of lesions**

To what extent do you assume that a full-body inspection has been performed by the general practitioner before starting the digital dermatoscopy consultation?* *Choose one of the following options:*

- I assume this has always been done
- I assume this has sometimes been done
- I assume this has not always been done

Do you consider the inability to perform a full-body inspection yourself a barrier for the digital dermatoscopy consultation?* *Choose one of the following options:*

- Yes
- No
- Please explain why you consider this a barrier or not

What is your preferred dermatoscopic technique when using digital dermatoscopy consultations?* *Choose one of the following options:*

- Contact dermatoscopy
- Polarized dermatoscopy
- Both

Where do you see opportunities for improvement in order to optimally assess a digital dermatology consultation?*

Other comments
