# Supplementary material for: An Exploration of Dutch Dermatologists’ Experience and Satisfaction With Teledermatology: Sociotechnical and Complex Adaptive System Perspective
Source: JMIR Dermatol. 2024 Jul 26;7:e56723. doi: 10.2196/56723 (PMC11316153; doi:10.2196/56723)
Supplement: Multimedia Appendix 2 [file derma_v7i1e56723_app2.docx]

**Appendix Table S1**. Codebook consisting of maincodes and subcodes

| **Maincode** | **Subcode** |
| --- | --- |
| General | DH+, Checking responsibility GP |
| Conditions for use | Choose patient, DC unnecessary, DC useful under C, DDS unsuitable, DH + for small group, DH difficult for C, DH limited in HC, Digital capable, enough pictures, P do not want DC, specialized GP |
| Effect on care | DC + effect on health, DC time efficient, Dh ensures less spread, DH limited contacts, DH no effect on health, do not use DH often, Less p to 2nd line |
| Userfriendliness | DC with other system, DH difficult for P, DH user unfriendly, DH userfriendly |
| Need for | DH + addition to HC, DH available for team, DH essential, DH good alternative, DH more applicable |
| Photo quality | Bad lightening picture, DDS Photo quality -, Improvement photo quality, No details on picture, No improvement photo quality, No picture by P, Photo quality -, Photo quality +, Photo quality other, Photo quality P -, Photo quality P +, Pictures blurry, Pictures sharp, To few pictures |
| Quality of care | Consultation with insufficient Information, Calling & pictures > DC, DC for short time, DC not replaces PC, DC partially replaces PC, DC replaces PC, Dermatoscopy risky, M assessment difficult, Missing dermatoscopy, Missing melanoma, Not always PC, Only 1 skin abnormality, P helped faster with DH, PC is needed, Photo quality > QoC |
| Training | Getting used to DH, No support needed, No support received, No useful support Ksyos, Online support DH, Picture/DH instruction, Support colleague, Visit for explanation |
| Workload | DC no effect WL, DC not lower WL |
| Learnability | Digital capable, learned DC, learning effect, no learning effect |
| System use | Back to normal after C19, C with insufficient Inf, Clear referral criteria, DC P sends pictures, DDC correct use, DDS final option, DDS for cancer, DDS for chronic patient, DDS for eldery people, DDS for pigmented laesi, DDS for rough estimate, DDS not for atypical naevi, DDS not for cancer, DDS not for complex cases, DDS not for inflammatory, DDS not for pigmented L, DDS unsuitable, Direct refer P to Derm, Just as often/Same DH, More DH due to C19, New layout +, No improvements DDC, Not more DC due to C19, Refer P to dermatologist, Suspicion malignancy, Suspicion melanoma, Urgent problems |
| System failure/improvement | Dh user unfriendly, Functionalities not in DH, Improving upload photo, Layout app outdated, Less change HCI, Linking DS -, Linking/Feedback report HIS -, New layout -, New phone/attachments, Problem request R, Response time -, To few pictures, Update questionnaire DC, Warning quality |
| Communication Ksyos | Financial agreement, do not know all options |
| Not applicable | no response, not applicable |

**Appendix Table S2.** Dimensions with corresponding maincodes and subcodes.

|  | | # |
| --- | --- | --- |
| **System measurement and monitoring** | | **63** |
| **Conditions for use** | | **2** |
|  | DDS unsuitabler | 1 |
|  | DH difficult for C | 1 |
| **Effect on care** | | **3** |
|  | DC +effect on health | 1 |
|  | DH ensures less spread | 1 |
|  | Do not use DH often | 1 |
| **General** | | **8** |
|  | Checking Resp GP | 1 |
|  | DH + | 7 |
| **Learnability** | | **3** |
|  | No Learningeffect - Covid | 3 |
| **Need for** | | **3** |
|  | DH good alternative | 1 |
|  | DH more applicable | 1 |
|  | DH no added value | 1 |
| **Quality of care** | | **22** |
|  | DC not replaces PC | 1 |
|  | DC partially replaces PC | 1 |
|  | Dermatoscopy risky | 3 |
|  | Missing melanoma | 2 |
|  | Only 1 skin abnormality | 15 |
| **System use** | | **19** |
|  | DDS final option | 1 |
|  | DDS not for cancer | 1 |
|  | DDS not for pigmented L | 3 |
|  | DDS unsuitable | 7 |
|  | Just as often/Same DH | 4 |
|  | No improvements DDC | 3 |
| **Userfriendliness** | | **1** |
|  | DC with other system | 1 |
| **Workload** | | **2** |
|  | DC not lower WL | 2 |
| **Clinical content** | | **49** |
| **Photo quality** | | **35** |
|  | C with insufficient Inf | 1 |
|  | DDS Photo quality - | 3 |
|  | Improvement photo quality | 1 |
|  | No details on picture | 1 |
|  | Photo quality - | 3 |
|  | Photo quality + | 3 |
|  | Photo quality other | 4 |
|  | Photo quality P - | 3 |
|  | Pictures blurry | 14 |
|  | Pictures sharp | 1 |
|  | Too few pictures | 1 |
| **Quality of care** | | **11** |
|  | C with insufficient Inf | 7 |
|  | DC not replaces PC | 1 |
|  | Missing dermatoscopy | 2 |
|  | Photo quality > QoC | 1 |
| **System failure/improvement** | | **2** |
|  | Too few pictures | 2 |
| **Workload** | | **1** |
|  | DC not lower WL | 1 |
| **Organizational policies and procedures** | | **41** |
| **Effect on care** | | **1** |
|  | DC +effect on health | 1 |
| **System use** | | **41** |
|  | DDS for cancer | 1 |
|  | DDS for chronic patient | 2 |
|  | DDS for pigmented laesi | 10 |
|  | DDS not for atypical naevi | 3 |
|  | DDS not for cancer | 4 |
|  | DDS not for complex casus | 1 |
|  | DDS not for inflammatory | 3 |
|  | DDS not for pigmented L | 7 |
|  | DDS unsuitable | 9 |
| **Quality of care** | | **1** |
|  | Not applicable | 1 |
| **People** | | **22** |
| **Conditions for use** | | **2** |
|  | Digital capable | 1 |
|  | Specialized GP | 1 |
| **General** | | **1** |
|  | Checking Resp GP | 1 |
| **Learnability** | | **2** |
|  | No Learningeffect | 2 |
| **Photo quality** | | **1** |
|  | No picture by P | 1 |
| **System use** | | **4** |
|  | DDS for eldery people | 3 |
|  | Just as often/Same DH | 1 |
| **Training** | | **11** |
|  | Picture/DH instruction | 11 |
| **Userfriendliness** | | **1** |
|  | DH difficult for P | 1 |
| **Workflow and communication** | | **21** |
| **Conditions for use** | | **2** |
|  | DH + for small group | 1 |
|  | DH difficult for C | 1 |
|  | Training | 1 |
|  | No support received | 1 |
| **Need for** | | **1** |
|  | DH essential | 1 |
| **Quality of care** | | **6** |
|  | DC not replaces PC | 4 |
|  | DC partially replaces PC | 1 |
|  | M assessment difficult | 1 |
| **System failure/improvement** | | **1** |
|  | Functionalities not in DH | 1 |
| **System use** | | **7** |
|  | DDS for rough estimate | 1 |
|  | DDS not for atypical naevi | 1 |
|  | DDS not for cancer | 2 |
|  | DDS not for pigmented L | 1 |
|  | Direct refer P to Derm | 1 |
|  | Not applicable | 1 |
| **Workload** | | **3** |
|  | DC not lower WL | 3 |
| **Human computer interface** | | **9** |
| **System failure/improvement** | | **4** |
|  | DH user unfriendly | 1 |
|  | Functionalities not in DH | 1 |
|  | New layout - | 2 |
| **System use** | | **2** |
|  | New layout + | 2 |
| **Userfriendliness** | | **3** |
|  | DH user unfriendly | 2 |
|  | New layout - | 1 |
| **Hardware and software** | | **5** |
| **System failure/improvement** | | **5** |
|  | DH user unfriendly | 1 |
|  | Functionalities not in DH | 3 |
|  | New phone/attachments | 1 |
| **External rules, regulations, and pressures** | | **2** |
| **System use** | | **2** |
|  | Back to normal after C19 | 1 |
|  | More DH due to C19 | 1 |
| **Not able to code** | | **35** |
| **NA** | | **32** |
|  | C with insufficient Information | 1 |
|  | No response | 26 |
|  | Not applicable | 7 |
| **Total** | | **247** |
