# Supplementary figures and images for: An Exploration of Dutch Dermatologists’ Experience and Satisfaction With Teledermatology: Sociotechnical and Complex Adaptive System Perspective
Source: JMIR Dermatol. 2024 Jul 26;7:e56723. doi: 10.2196/56723 (PMC11316153; doi:10.2196/56723)

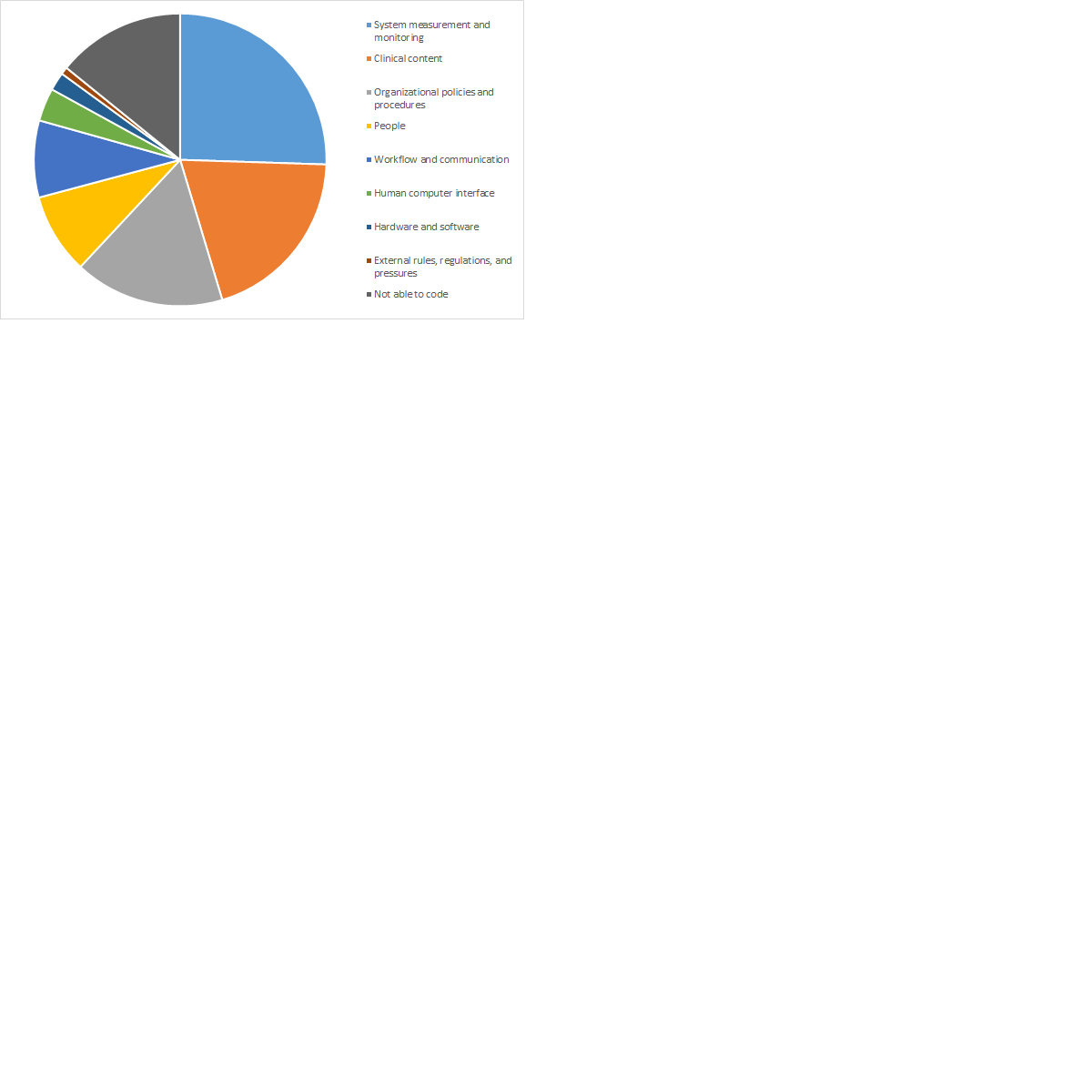

Supplement: Multimedia Appendix 3 [file derma_v7i1e56723_app3.png]
